# PRE-OPERATIVE FASTING: WHY ABBREVIATE?

**DOI:** 10.1590/0102-672020180001e1377

**Published:** 2018-07-02

**Authors:** Samara Bomfim Gomes CAMPOS, João Araújo BARROS-NETO, Glaucevane da Silva GUEDES, Fabiana Andréa MOURA

**Affiliations:** 1 Faculdade de Nutrição, Universidade Federal de Alagoas, Maceió, AL, Brazil

**Keywords:** Fasting, Carbohydrates, Preoperative care, Jejum, Carboidratos, Cuidados pré-operatórios

## Abstract

*****Introduction*** ****:**

Considering the practice of preoperative fasting based on observations on the gastric emptying delay after induction and the time of this fast is closely linked to organic response to trauma, arise the question about preoperative fasting period necessary to minimize such response and support the professional with clinical and scientific evidence.

*****Aim*** ****:**

To review the aspects related to the abbreviation of preoperative fasting from the metabolic point of view, physiology of gastric emptying, its clinical benefits and the currently recommendations.

*****Method*** ****:**

Literature review was based on articles and guidelines published in English and Portuguese, without restriction of time until January 2017, in PubMed, SciELO and Cochrane with the descriptors: surgery, preoperative fasting, carbohydrate. From the universe consulted, 31 articles were selected.

*****Results*** ****:**

The literature suggests that the abbreviation of fasting with beverage added carbohydrates until 2 h before surgery, can bring benefits on glycemic and functional parameters, reduces hospitalization, and does not present aspiration risk of healthy patients undergoing elective surgery. Another nutrient that has been added to the carbohydrate solution and has shown promising results is glutamine.

*****Conclusion*** ****:**

The abbreviation of preoperative fasting with enriched beverage with carbohydrates or carbohydrate and glutamine seems to be effective in the care of the surgical patient, optimizing the recovery from of postoperative period.

## RESUMO

*****Introdução*** ****:**

Considerando que a prática do jejum pré-operatório é baseada nas observações do retardo do esvaziamento gástrico após a indução anestésica e que o tempo deste jejum está intimamente ligado à resposta orgânica ao trauma, surge o questionamento acerca do período de jejum necessário para minimizar tal resposta e respaldar a atuação profissional em evidências clinicocientíficas.

*****Objetivo*** ****:**

Revisar a abreviação do jejum pré-operatório, sob o ponto de vista metabólico, da fisiologia do esvaziamento gástrico, seus benefícios clínicos e recomendações atualmente vigentes.

*****Método*** ****:**

A revisão foi baseada em artigos e guidelines publicados em inglês e português, sem restrição de tempo até janeiro de 2017, no PubMed, SciELO e Cochrane com os descritores: cirurgia, jejum pré-operatório, carboidrato. Do universo consultado 31 artigos foram selecionados.

A literatura aponta que a abreviação do jejum com bebida enriquecida com carboidratos até 2 h antes do procedimento cirúrgico pode trazer benefícios sobre parâmetros glicêmicos, funcionais, redução da hospitalização, além de não oferecer risco de broncoaspiração em indivíduos saudáveis submetidos à operações eletivas. Outro nutriente frequentemente adicionado a esta solução de carboidratos, com resultados promissores, é a glutamina.

*****Conclusão*** ****:**

A abreviação do jejum pré-operatório com bebida enriquecida com carboidratos ou carboidratos e glutamina parece mostrar-se eficaz no cuidado ao paciente cirúrgico otimizando a recuperação do período pós-operatório.

## INTRODUCTION

Preoperative nocturnal fasting was instituted when anesthetic techniques were still quite rudimentary, and chloroform was used at the time^18^ and its main objective was to avoid respiratory complications due to vomiting and aspiration of gastric contents^25^. These recommendations were based on the symptoms described in Mendelson’s syndrome - in honor of the American obstetrician, who in 1946 reviewed cases of death in pregnant women related to the aspiration of solid gastric contents in operations with general anesthetic induction. From these observations, gastric emptying delay was postulated during labor and the “nothing by mouth” recommendation was generated before induction of anesthesia, with the establishment of nocturnal preoperative fasting^10^ ^,^ ^25^.

With the advent of evidence-based medicine, the need arises to base clinical practices from old paradigms or empirically conceived, thus directing clinical studies that scientifically ground new therapeutic strategies^10^ ^,^ ^16^.

Considering the prolonged fasting time to which patients are frequently submitted, as well as the metabolic and clinical losses associated with this practice, leading to implications on the quality of life and general well-being of the surgical patient, there is a need to elucidate the several factors associated with prolonged fasting, as well as strategies to reduce their time, in order to minimize the deleterious effects of the organic response to trauma^3^ ^,^ ^12^ ^,^ ^28^.

Thus, the present study aims to review the aspects related to the abbreviation of metabolic preoperative fasting, the physiology of gastric emptying, its clinical benefits, as well as the recommendations currently in force.

## METHODS

It is a narrative review of literature based on articles and guidelines published in English and Portuguese, without restriction of time until January 2017. The search included PubMed, SciELO and Cochrane databases with the descriptors: surgery, preoperative fasting and carbohydrate. When necessary, the strategy was adapted to the databases used. Of the universe consulted, 31 articles were selected. Also were included printed materials available to the researchers and articles identified with this theme, but not filtered by the search method initially defined for this research, thus characterizing a non-systematic narrative review.

## RESULTS

### Metabolic implications of fasting on surgical trauma

Organic homeostasis is finely regulated for maintenance at basal levels. When a person undergoes the fasting process, several reactions occur in order to maintain glycemic and energy supply. To this end, it uses glycogenolysis metabolic cascades, proteolysis - to obtain gluconeogenic substrates - and lipolysis, which also involve hormonal alterations such as glucagon circulation^21^ ^,^ ^22^.

The organic response associated with the trauma/surgical procedure is defined as a multifactor physiological response, where the long fasting period added to the trauma imposed by the operation implies an increase in catabolic hormones such as cortisol and glucagon, inflammatory response and catecholamine secretion. The increase of these hormones results in insulin resistance with a very similar characteristic to that observed in type 2 diabetes, where the uptake of glucose by the cells is diminished by the inability of the glucose transporter type 4 (GLUT-4) to perform such action. The main consequence is a state characterized by catabolism, which includes the high consumption of glycogen reserves, with a reduction of its synthesis, and also proteolysis and lipolysis^12^ ^,^ ^21^ ^,^ ^22^.

 Despite the knowledge regarding the organic response associated with the trauma and the metabolic implications of prolonged fasting, in clinical practice the fasting time practiced is around 12 h^3^, exceeding even the traditionally instituted night fast of 8 h. This higher food deprivation time correlates with important clinical implications such as hunger sensation, thirst, longer hospitalization time, surgical site infection, operative complications and death^7^ ^,^ ^12^ ^,^ ^22^ ^,^ ^28^.

Thus, it is important to note that prolonged fasting, whose main objective is to avoid respiratory complications due to vomiting and aspiration, secondary to a possible delayed or insufficient gastric emptying, finds other complicating factors, clinical and metabolic, that can put at risk the postoperative recovery of patients.

### Physiology of gastric emptying and safety of fasting abbreviation

Several factors influence gastric emptying. However, what has the greatest influence on this physiological process is the quantity and composition of the chyme that reaches the duodenum^8^. In this context, through a negative feedback mechanism mediated by cholecystokinin, gastric emptying is inhibited as chyme reaches the duodenum, especially when it has a higher lipid content^8^ ^,^ ^20^.

The literature indicates that solutions with the same amount of carbohydrate (50 g), but with different volumes (300-400 ml), have similar gastric emptying rates, suggesting that this process depends to a large extent on the presence of nutrients than necessarily the volume, osmolarity, density or viscosity of the solution^23^.

Regarding the interference of gender on the velocity of emptying, despite the old knowledge of the effect of gender hormones on mucosal motility^11^, these do not seem to affect the rate of emptying of clear liquids, and no significant differences were observed between genders^23^.

Another point of considerable discussion is the body mass factor. It was observed that the gastric content did not differ between obese patients (BMI> 30 kg/m^2^) who ingested 300 ml of clear liquids 2 h before the operation when compared to those submitted to conventional fasting, both of whom underwent elective operation under general anesthesia^24^. Despite the findings, to date, the recommendations for preoperative fasting do not apply to obese individuals^4^, considering the increased risk of bronchoaspiration and the interference of body mass factor on gastric emptying^24^. Thus, these individuals are submitted to the protocol of traditional nocturnal fasting^4^.

Considering the multiple factors involved in the gastric emptying process, research methods have been conducted with the objective of elucidating the safety of the abbreviation of fasting through the use of imaging techniques such as magnetic resonance^9,23^ and scintigraphy^5^. The results demonstrate that residual gastric volume returns to baseline levels after 120 min of ingestion of a carbohydrate solution (50 g dissolved in 400 ml)^23^ because it is rapidly emptied by the stomach, given the regulatory and integrative mechanisms between the stomach and the intestine^4^ ^,^ ^5^ ^,^ ^7^ ^,^ ^17^ ^,^ ^26^. In the study by Lobo et al.^23^, was evaluated gastric emptying for beverages added with carbohydrates (50 g), glutamine (15 g), vitamin C (750 mg), vitamin E (250 mg), green tea extract, ß-carotene (5 mg), zinc (10 mg) and selenium (150 μg), diluted in 400-300 ml, and was observed that the residual gastric volume returned to its basal levels after 180 min in healthy people^23^.

Additionally, some specific conditions were evaluated regarding the safety of the fasting abbreviation with the use of carbohydrate solution^1^ ^,^ ^17^. Aguilar-Nascimento et al.^1^ when evaluating the effect of the ingestion of 200 mL of carbohydrate beverage at 12.5% ​​offered 2 h prior to laparotomic cholecystectomy, did not report any infectious complications or deaths among the patients in the test group. Already Hausel et al.^17^ evaluating individuals with indication of elective abdominal surgery, distributed 252 subjects in three subgroups: test group or fasting, who received 800 ml of carbohydrate solution (12.5%) the night before the operation and 400 ml of the same solution, up to 2 h prior to premedication on the morning of the procedure; placebo group, which received flavored water at the same times and volumes of the abbreviated fasting group; and the control or fasting group, which underwent the traditional nocturnal fasting period. According to these authors, there was no increase in gastric contents or changes in stomach pH in patients submitted to the abbreviation of fasting^1^ ^,^ ^17^.

The result of a recent meta-analysis conducted by Awad et al^.6^ with 1,685 non-diabetic adult patients, found that providing oral solution with 50 g of carbohydrates, offered 2-4 h prior to the surgical procedure, appears to be safe for the abbreviation of fasting preoperative period, with no complications recorded^6^.

### Benefits of abbreviation of preoperative fasting

Several studies using the carbohydrate fasting protocol exclusively, or with the combination of these with glutamine, demonstrate reductions in glycemia, insulinemia and insulin resistance^5^ ^,^ ^6^ ^,^ ^27^.

The decrease in insulin resistance after the use of carbohydrate solutions is possibly due to the ability of glucose to modulate the catabolic and inflammatory response inherent to surgical trauma, improving insulin sensitivity^12^ ^,^ ^13^ ^,^ ^29^.

The abbreviation of the preoperative fast also contributes to the reduction in the length of hospital stay, as demonstrated by Feguri et al.^15^. According to these authors, patients who underwent myocardial revascularization and who received a solution with maltodextrin (12.5%), 6 h (400 ml) and 2 h (200 ml) before the surgical procedure, reduced the hospitalization and in one day the length of stay in the intensive care unit. A justification for this finding would be the best insulin response found in the group that received the carbohydrate solutions, resulting in greater glycemic control, a condition closely linked with greater clinical severity and, therefore, a longer hospital stay^15^ ^,^ ^17^.

Still in this context, the ingestion of carbohydrate and glutamine solution, offered in the preoperative period, seems to favor even more the postprandial glycemic control evidenced by the reduction of glucose and insulin^5^. Dock-Nascimento et al.^12^ observed that consumption of 400 ml of a carbohydrate and glutamine drink, 8 h (50 g of maltodextrin + 40 g of glutamine) and 2 h (25 g of maltodextrin + 10 g of glutamine) before surgical procedure, resulted in several benefits, such as: reduction in the acute response phase; improvement of antioxidant defenses, with increased levels of glutathione; and reduced levels of cortisol, catabolic hormone intrinsically related to the response to trauma, and which leads to elevated glycemic levels, lipolysis and proteolysis. In addition, there was an improvement in the nitrogen balance, suggesting the preservation of muscle mass in the postoperative period^12^.

The benefits of fasting abbreviation appear to be more intense in individuals undergoing a large surgical procedure, since the picture of insulin resistance is proportional to the surgical trauma. In this way, the insulinemic and glycemic control would contribute more decisively to the improvement of the clinical response of the patient^5^ ^,^ ^6^ ^,^ ^27^.

With regard to well-being and comfort, the literature shows that the abbreviation of fasting can significantly reduce the sensation of hunger, thirst, dry mouth, nausea, and weakness^28^. Regarding the occurrence of gastrointestinal symptoms, the study by Aguilar-Nascimento et al.^1^ concluded that abdominal distension, vomiting and the association of two or more symptoms involving the gastrointestinal tract were significantly lower among patients receiving carbohydrate preoperativelly1.

The preoperative fasting time also influences functional parameters, such as the strength of the palmar grip determined by dynamometry, an indicator of postoperative complications related to worsening of functional status during hospitalization^19^ ^,^ ^31^. In a study conducted by Zani et al.^31^ patients who received carbohydrate solution, 6 h (400 ml) and 2 h (200 ml) before the surgical procedure containing 50 g and 25 g of maltodextrin, respectively, presented higher values ​​of the strength of palmar grip, both in the dominant and in the non-dominant hand. According to these authors, the group that received the intervention showed a significant improvement in respiratory function, assessed by the peak of expiratory flow in the first second and greater forced vital capacity, in comparison to the group that remained fast. In addition, the fasting with carbohydrate-enriched drink (50 and 25 g of CHO, 8 h and 2 h before operation in 400 and 200 ml, respectively) or carbohydrate in the same amounts, added with 40 and 10 g of glutamine in each outcome, presented a relation with lower inflammatory activity, confirmed by the lower C-reactive protein (CRP)/albumin ratio^12^.

### Recommendations for fasting abbreviation

Considering the organic response to trauma and the optimization of surgical patient recovery, the American Society of Anesthesiologists (ASA), the world reference in anesthesiology, makes its recommendations for the preoperative period more flexible, with the recommendation of anticipation of fasting through the ingestion of clear liquids up to 2 h and light meals (without fried foods, fatty foods or meat) in up to 6 h for healthy patients, before elective surgical procedures that require general anesthesia, local or sedation/ analgesia^4^. Exception is made to patients with coexisting conditions or conditions that affect gastric emptying and volume, such as gestation, diabetes, obesity, hiatal hernia, gastroesophageal reflux disease, intestinal obstruction, emergency operations, enteral tube feeding, and patients in whom the management of the airways is difficult^4^.

The European ERAS (Enhanced Recovery After Surgery) protocol of the European Society of Clinical Nutrition and Metabolism, which uses multidisciplinary actions to reduce the stress associated with trauma, thus enabling faster recovery after a large operation, also recommends reduction in preoperative food deprivation, indicating fasting of 2 h for liquids and 6 h for solids, with the supply of carbohydrate-containing fluids and beverages^14^.

In Brazil, the program entitled ACERTO (Acceleration of Total Post-Operative Recovery) was designed with the objective of accelerating the recovery of patients in the postoperative period. Its implantation occurred in the Department of Surgical Clinic of the Júlio Muller University Hospital of the Federal University of Mato Grosso, MT, Brazil in 2005, with the participation of the multidisciplinary health team. This program considers several aspects of surgical patient care, from venous hydration and antibiotic therapy to perioperative nutrition. In the latter, an abbreviation protocol for fasting recommends the administration of a carbohydrate solution (maltodextrin) at 12.5%, given 6 h and 2 h before the surgical procedure^2^.

Despite the recommendations, the implantation of these protocols is still incipient in the country, as can be seen in the multicenter study (16 hospitals in nine states of the country) performed by Aguilar-Nascimento et al. (2014)^3^. Among the main results, the high time (6-8 h) of preoperative fasting performed in most hospitals (75%) stands out. In addition, it was found that food deprivation is often greater than prescribed, since almost 80% of the patients have their operation performed after 8 h of fasting. According to these authors, among the possible causes of the long fasting period are delays in the schedules of the surgical procedure, changes in the scale of operations and the length of fasting prescribed by the patients themselves, believing that this would improve their response to the procedure^3^.

## CONCLUSION

Fasting preoperative night-time “nothing by mouth”, often practiced, does not seem to be the best preparation option for candidates for elective surgery from the metabolic point of view and the patient’s own well-being. In turn, several benefits such as better glycemic control and shorter hospitalization time are achieved through fasting with a carbohydrate-rich beverage offering up to 2 hours before the surgical procedure, and this practice is recommended for all elective patients, with exceptions that affect gastric emptying. In addition, carbohydrate solutions with glutamine appear to be promising for improving postoperative metabolic response.
